# Improving Hand Hygiene Compliance in Nursing Homes: Protocol for a Cluster Randomized Controlled Trial (HANDSOME Study)

**DOI:** 10.2196/17419

**Published:** 2020-05-01

**Authors:** Gwen R Teesing, Vicki Erasmus, Mariska Petrignani, Marion P G Koopmans, Miranda de Graaf, Margreet C Vos, Corné H W Klaassen, Annette Verduijn-Leenman, Jos M G A Schols, Jan Hendrik Richardus, Helene A C M Voeten

**Affiliations:** 1 Department of Public Health Erasmus MC, University Medical Center Rotterdam Rotterdam Netherlands; 2 Municipal Public Health Service Rotterdam-Rijnmond Rotterdam Netherlands; 3 Municipal Public Health Service Haaglanden Den Haag Netherlands; 4 Municipal Public Health Service Amsterdam Amsterdam Netherlands; 5 Department of Viroscience Erasmus MC, University Medical Center Rotterdam Rotterdam Netherlands; 6 Department of Medical Microbiology and Infectious Diseases Erasmus MC, University Medical Center Rotterdam Rotterdam Netherlands; 7 Pieter van Foreest Delft Netherlands; 8 Department of Health Services Research and Department of Family Medicine Maastricht University Maastricht Netherlands

**Keywords:** hand hygiene, protocol, nurse, nursing home, randomized controlled trial, intervention

## Abstract

**Background:**

Hand hygiene compliance is considered the most (cost-)effective measure for preventing health care–associated infections. While hand hygiene interventions have frequently been implemented and assessed in hospitals, there is limited knowledge about hand hygiene compliance in other health care settings and which interventions and implementation methods are effective.

**Objective:**

This study aims to evaluate the effect of a multimodal intervention to increase hand hygiene compliance of nurses in nursing homes through a cluster randomized controlled trial (HANDSOME study).

**Methods:**

Nursing homes were randomly allocated to 1 of 3 trial arms: receiving the intervention at a predetermined date, receiving the identical intervention after an infectious disease outbreak, or serving as a control arm. Hand hygiene was evaluated in nursing homes by direct observation at 4 timepoints. We documented compliance with the World Health Organization’s 5 moments of hand hygiene, specifically before touching a patient, before a clean/aseptic procedure, after body fluid exposure risk, after touching a patient, and after touching patient surroundings. The primary outcome is hand hygiene compliance of the nurses to the standards of the World Health Organization. The secondary outcome is infectious disease incidence among residents. Infectious disease incidence was documented by a staff member at each nursing home unit. Outcomes will be compared with the presence of norovirus, rhinovirus, and Escherichia coli on surfaces in the nursing homes, as measured using quantitative polymerase chain reaction.

**Results:**

The study was funded in September 2015. Data collection started in October 2016 and was completed in October 2017. Data analysis will be completed in 2020.

**Conclusions:**

HANDSOME studies the effectiveness of a hand hygiene intervention specifically for the nursing home environment. Nurses were taught the World Health Organization’s 5 moments of hand hygiene guidelines using the slogan “Room In, Room Out, Before Clean, After Dirty,” which was developed for nursing staff to better understand and remember the hygiene guidelines. HANDSOME should contribute to improved hand hygiene practice and a reduction in infectious disease rates and related mortality.

**Trial Registration:**

Netherlands Trial Register (NTR6188) NL6049; https://www.trialregister.nl/trial/6049

**International Registered Report Identifier (IRRID):**

DERR1-10.2196/17419

## Introduction

Health care–associated infections (HAI) are a significant source of morbidity in nursing home residents. If we include urinary tract infections, we see on average more than one HAI per resident per year in European nursing homes [1]. Not only do residents become ill from HAI but HAI may also affect staff due to their own illness and increased workload, further disrupting care. Hand hygiene (HH) can play a role in an infection prevention strategy.

Most studies focus on hand hygiene compliance (HHC) in hospitals, ignoring other settings with vulnerable populations, such as nursing homes [2]. The few published studies that recorded HHC in nursing homes according to the World Health Organization (WHO) standards show estimates of 6% to 27% HHC before an intervention [3-7]. There is some evidence that infectious disease rates and mortality rates decrease in nursing homes when HHC increases through HH interventions [4,8-10]. While most HH intervention studies document HHC rates in hospitals, there are a few published studies showing that interventions can significantly influence HHC in a nursing home [4,8,11]. For example, 2 studies in long-term care facilities in Hong Kong showed significant increases in HHC in intervention arms (27% to 61%, *P*<.001; 22% to 49%, *P*<.001; and 26% to 33%, *P*=.10), no significant changes in control arms after implementing multifaceted HH interventions involving the provision of hand sanitizer, reminder materials, education, and, in one case, performance feedback [4,8]. In Taiwan, nursing assistants showed significantly better HHC (from 9% to 30%, *P*<.001) 3 months after participating in a 1-hour class and 30 minutes of hands-on training [11].

Due to a paucity of HH studies in nursing home settings using the WHO hand hygiene standards, we designed a trial to evaluate the impact of an intervention package tailored to the specific context of nursing homes. HH interventions developed for hospitals are not necessarily appropriate for nursing homes. First, the 5 HH moments of the WHO are difficult to interpret and use in the nursing home setting. The 5 moments of the WHO dictate that HH should be done before touching a patient, before a clean/aseptic procedure, after body fluid exposure risk, after touching a patient, and after touching patient surroundings. At the same time, a patient’s surroundings in a nursing home is a fluid concept. Nursing home residents are generally mobile, sharing communal areas. For example, should touching a resident’s walking frame in the living room be considered touching a resident’s environment (after which HH is indicated)? Is a section of a table in a living room a particular “resident’s environment” because that resident is sitting there at that moment? Second, interventions should minimally disturb the homelike setting. For example, hanging hand sanitizer dispensers on beds could be perceived as transforming the homelike environment to a medicalized one. Another difference is that nurses in nursing homes generally have less education than nurses who work in hospitals. The intervention should therefore be adapted to their educational level by using simple language and hands-on exercises [12].

The HANDSOME study was developed to evaluate the effectiveness of an intervention to improve HHC in nursing homes. An additional goal of the study is to determine if an intervention is more effective when implemented following an outbreak. In this paper, we describe the study design and protocol details of the HANDSOME study.

## Methods

### Overview

The HANDSOME intervention is based on our experience with developing HH interventions in hospital and childcare settings. We performed a randomized controlled HH study in 15 hospitals throughout the Netherlands [13]. Underlying determinants for HHC were addressed through various means, including making changes to the physical environment (eg, adding dispensers), creating new social norms, and implementing an HH e-Learning program. While the control and intervention arms did not differ in HHC at baseline, there was a statistically significant difference in HHC during the follow-up between the control arm (24.9% HHC) and intervention arm (35.4% HHC) [14]. In childcare settings, we conducted a cluster randomized controlled trial including providing HH products, providing HH training to childcare workers, organizing team sessions to promote goal setting, and providing stickers and posters for caregivers and children as cues to action. This led to a statistically significant increase in HHC in the intervention arm, even 6 months after the intervention [15]. Considering the significant increases in HH in these settings, we adapted these interventions for the current study.

### Trial Design

HANDSOME is a parallel-group, observer-blinded, and observed-blinded cluster randomized controlled trial to increase nurses’ HHC. For the purpose of this study, nurses were defined as those who have completed a 3-year or 4-year degree in nursing. The study has 3 study arms: 2 intervention arms and 1 control arm. Nursing homes were randomized to one of the 3 trial arms: fixed intervention, conditional intervention, and control. The nursing homes in the 2 intervention arms received the same intervention, while the control nursing homes did not receive the intervention. The nursing homes in the fixed intervention arm received the intervention at a predetermined date, while the nursing homes in the conditional intervention arm received the same intervention as the fixed intervention arm, but only after an infectious disease outbreak. The conditional intervention arm was conceived with the idea that an outbreak would cause an increased sense of infection risk and urgency, leading towards a better and/or more sustained HH performance. The control locations were free to implement any other infection prevention intervention, since this is “business as usual” and it is unethical to withhold care improvements from residents. Nursing homes were observed several times for HHC, required to complete illness incidence reports, and subjected to microbiological surface sampling.

Background information about the nursing homes was collected through a structured interview. This was followed by a baseline observation in every nursing home unit. Next, nursing homes were randomized into 1 of the 3 study arms. Randomization was at the level of the nursing home rather than the individual nurse or ward, since the intervention was available to an entire nursing home. The aim was to include a minimum of 55 nursing homes: 15 fixed intervention nursing homes, 25 conditional intervention nursing homes, and 15 control nursing homes (see Sample Size Calculations).

A tablet-based app was used to document compliance. Results from background interviews, pilot observations, and the pilot intervention were used to refine the observation app and intervention. Since we were able to determine which types of HH opportunities (submoments) are the most common, these were added to the app to get more insight into HHC. We also used this extra information to address specific HH issues during the intervention, such as how to handle laundry or use a telephone, tablet, or hand brace. We were also able to specifically incorporate the most common invasive procedures in the intervention lessons. The pilot intervention allowed us to revise the materials so that they were easier to use.

### Trial Aim

We aimed to increase compliance with the WHO’s 5 moments of HH [16], which was measured during repeated observations over a period of 12 months.

### Study Setting

All data were collected in nursing homes in the Netherlands. To capture diversity, these nursing homes are situated throughout the country in areas with differing degrees of urbanization.

### Recruitment

Recruitment of nursing homes began by sending printed flyers with information about the study to large nursing home organizations listed on a website that lists most health care providers in the Netherlands (*ZorgkaartNederland*). Digital flyers were also sent to health care associations so they could inform their members about the study. In addition to the nursing homes recruited for the study, 3 nursing homes from 3 distinct organizations were recruited as pilot locations to train observers and test the intervention. After the distribution of the flyers, organizations were contacted by phone to discuss willingness, eligibility, and conditions for participation. Interested nursing homes were visited personally to further discuss participation. Enrollment began April 25, 2016. Participants are no longer being recruited.

### Eligibility Criteria

Eligibility criteria were identified to foster homogeneity between nursing home units. First, only publicly funded organizations willing to commit 3 or 4 nursing homes to the study were eligible. By allocating different nursing homes within the same organization to different study arms, we aimed to minimize variation between the study arms. Each nursing home committed a minimum of 2 eligible units. Nursing home wards were considered eligible as a unit if they had 3 or more nurses working between 8:00 am and 2:00 pm on weekdays so that we could observe a minimum of 3 nurses during one observation session. If there were not enough nurses employed during those hours in one ward, multiple wards were combined and considered 1 unit for purpose of this study. If a nursing home could only supply 1 unit, it was coupled with a unit from another nursing home from the same organization. All wards primarily provided somatic or psychogeriatric residential care. Nursing homes were allowed to perform other infection prevention improvements, provided they did not simultaneously participate in other HH trials.

### Allocation

The randomization process was accomplished through a stepwise procedure after baseline observations. The primary investigator first drew (computer-generated) one nursing home per organization at random for the fixed intervention arm. After this, one nursing home per organization was randomly drawn for the conditional intervention arm. The remaining nursing homes were randomly assigned to the conditional intervention arm or the control arm. This method allowed for random allocation while minimizing the variation between the study arms.

### Intervention

Studies have shown that using multiple strategies that address multiple determinants (eg, a multimodal approach) is the most effective in increasing HHC [17]. Another key determinant for good HHC is repetition [17-19]. These were the cornerstones of our intervention.

For the purpose of the current trial, we scanned literature for determinants that influence HH [18,20,21], in particular for determinants that we had not considered in our earlier interventions. Additionally, 5 interviews were held at nursing homes for a better understanding of obstacles to HH. Next, intervention mapping principles were used to further recognize applicable determinants, methods, and strategies for the development of this intervention [22] (Table 1). The intervention was further refined after informal discussions with members of more than 20 nursing home organizations during the recruitment period. The intervention continued to be adjusted as an iterative process.

**Table 1:** Intervention mapping for HANDSOME: using determinants and methods to develop the strategy for intervention components.

Intervention element		Determinant(s)	Method(s)/strategy(s)
**Meeting with management**			
	Present the average HHC^a^ in nursing homes. Show there is room for improvement.	Knowledge	Reporting
	Talk about costs (time and money) and harm (illness of residents and staff) associated with a methicillin-resistant *Staphylococcus aureus* or norovirus outbreak.	Perceived threat, acknowledging importance	Consciousness raising, persuasive communication, anticipated regret
	Use a form to structurally discuss necessary facilities and facility changes for efficient HH^b^ practices. Stress that the organization, not the resident, must provide all HH materials. Help optimize where HH materials are stored and how and when they are replaced.	Environmental restructuring, rules and regulations, awareness, assistance for organizational change	Organizational diagnosis and feedback/tailoring, systems change, reduce environmental barriers, persuasive communication, participatory problem solving, structural redesign, cue altering/nudging, consciousness raising, goal setting, problem management tool
	Talk about the Dutch guidelines for personal hygiene and noncompliance policies at other organizations. Talk about risk of infection. Use a form to register a (new) personal hygiene policy for the organization. Make sure that employees have a safe space for personal belongings. Offer solutions for personnel with rings.	Seeing importance, rules and regulations, professional standards	Systems change, nonfinancial incentives, mandate, anticipated regret, tailoring, organizational diagnosis tool
	Let management know that they can receive a “Good hand hygiene” certification if they achieve a minimum HHC.	Motivation	Nonfinancial incentives, early commitment
	Convince management that their presence at Lesson 1 will positively influence HHC results. Plan lessons and the personal hygiene presentation.	Capable leadership	Persuasive communication (with management), planning
**Lesson 1**			
	A senior nursing home manager introduces the intervention and expresses the importance of HH.	Leadership commitment, framing	Persuasive communication, public commitment, introduce systems change
	Show an HH video. Present health care–associated infection statistics for nursing homes and explain health risk to self and others. Help employees visualize HH from the perspective of the resident.	Create urgency, framing	Persuasive communication, consciousness raising, anticipated regret, shifting perspective
	Teach using a presentation. Teach “Room In, Room Out, Before Clean, After Dirty.” Teach and discuss HH when handling pills, food, and laundry. Teach when to use hand sanitizer or soap and the proper use of gloves.	Knowledge	Chunking, using imagery, personal feedback
	Team creates a group HH goal.	Self-efficacy, sense of ownership	Implementation intentions/goal setting, social influence, team commitment
	Introduce the e-Learning and show the nurse’s watch they can earn by completing the e-Learning.	Facilitate learning, nonfinancial incentives	Structural redesign, beginning of repeated exposure
	Show posters and ask where they want to see the posters. Hand out small bottles of hand sanitizer for use in the e-Learning,	Nonfinancial incentives, self-efficacy, sense of ownership	Cue altering
**Presentation of the personal hygiene policy**			
	A senior nursing home manager presents the personal hygiene policy (no long nails, nail polish, rings, bracelets, watches, braces, or long sleeves). Make consequences known for noncompliance.	Mandate, perceptions of norms, leadership commitment	Punishment, persuasive communication, role models
**Lesson 2**			
	Make an inventory of barriers to good HH.	Attitude, knowledge	Discussion
	Think of solutions for barriers.	Systems change	Tailoring, organizational diagnosis, planning coping responses, group discussion, structural redesign, systems change
**Lesson 3**			
	Participants “wash” hands with paint and see where they miss.	Attitude, knowledge	Participation
	Participants learn how to disinfect their hands.	Knowledge, self-efficacy	Guided practice
	Participants see that they get paint on hands after glove removal and that the paint represents invisible bacteria/viruses.	Knowledge	Anticipated regret, rationalize risk
	Remind participants that they can earn a watch by completing the e-Learning.	Non-financial incentives	Persuasive communication
**E-Learning**			
	Show playback squelching excuses not to do HH. Show films from the perspective of the resident.	Professional behavior standards, attitude	Using imagery, shifting perspective
	Explain when to use hand sanitizer or soap. Practice using hand sanitizer with participants.	Knowledge, skills, self-efficacy	Advance organizers, modelling, guided practice
	Use videos with correct and incorrect behavior to teach HH moments and common HH actions. Teach how to perform HH when preparing food and pills.	Knowledge	Chunking, modelling, active learning
	Teach how to work efficiently to avoid unnecessary HH using videos with correct and incorrect behaviors.	Clinical work process flow	Systems change
	Teach the proper use of gloves with still images and videos with correct and incorrect behaviors.	Perceived norms, knowledge	Active learning, imagery, modelling, persuasive communication
	Show that HH does not inhibit other tasks or social contact with the resident.	Self-efficacy	Modelling
	Give a quiz after every module.	Knowledge	Reinforcement through testing, feedback, monitoring
	Promise a nurse’s watch when the e-Learning is completed. Use dripped learning so that the e-Learning is completed in small modules over 14 weeks. Send reminders.	Curiosity, information system, knowledge, nonfinancial incentive	Facilitation, anticipated regret, reminders, repetition
**Poster**			
	Multiple copies of a new poster are hung throughout the nursing home every month.	Social influence, perceived norms	Visuals, repeated exposure, cue to action
**Photo competition**			
	Let nursing home employees know they can win a prize for the best photo of hands.	Nonfinancial incentives	Providing cues
**Arts and crafts project**			
	Residents are informed about HH and the organization’s HH goals.	Knowledge	Consciousness raising
	Residents perform an activity involving hands. Nursing home displays artwork.	Perceived norms	Participation, cues to action

^a^HHC: hand hygiene compliance.

^b^HH: hand hygiene.

The intervention has 4 main components: a meeting with the management, 3 live group lessons, e-Learning, and posters. Additionally, there is a photo competition and an arts and crafts project. All components were published on a website after completion of the intervention [23].

#### Meeting With Management

A meeting at the nursing home took place 1-2 months after the baseline compliance measurement. A senior nursing home manager, infection prevention specialist, and facilities manager were asked to attend the meeting. The meeting started with consciousness raising about the cost of a methicillin-resistant *Staphylococcus aureus* (MRSA) outbreak so the participants would anticipate regret if they did not implement necessary changes. Next, information about the intervention and necessary facilities for HH were presented. Removing environmental barriers and adding cues to action were discussed, including the strategic placement of hand sanitizer and posters. Tailored system changes were advised to encourage better HH, such as how to hygienically dispose of dirty laundry.

The Dutch guidelines for (hand-related) personal hygiene dictate that staff members providing care do not wear rings, nail polish, artificial nails, long nails, bracelets, watches, a brace, or long sleeves [24]. Policy changes for personal hygiene noncompliance were discussed, including disciplinary consequences. Management was also asked to give a personal hygiene presentation between the first and second lesson. Although personal hygiene is broader than hand-related personal hygiene, we stressed the need to address hand-related personal hygiene.

Nursing homes were also promised a nonfinancial incentive. If they had a higher than average HHC, they would receive a certificate of good HH. At the end of the meeting, an intervention implementation schedule was discussed. While the compliance measurements were only completed at certain wards, all nurses and nurses’ aides from the entire nursing home were welcome to participate in the intervention.

#### Lessons

A member of the study team provided 3 lessons lasting a half hour each. The lessons were generally given multiple times on one day to a maximum of 18 participants per session.

The first lesson began with an introduction by a senior nursing home manager, showing leadership commitment to systems change. The first goal of the lesson was to create awareness about the necessity of HH. Still images, video, and a persuasive live presentation promoted consciousness raising and anticipated regret. The second goal was to teach the participants when they needed to perform HH. They were taught using a novel description of the 5 HH moments of the WHO [25], namely “Room In, Room Out, Before Clean, After Dirty” (“Kamer in, Kamer uit, Voor schoon, Na vies”). “Room In” corresponds to the WHO Moment 1 (before touching a patient). “Room Out” corresponds to WHO Moment 4 (after touching a patient) and Moment 5 (after touching patient surroundings). “Before Clean” corresponds to WHO Moment 2 (before a clean/aseptic procedure), and “After Dirty” corresponds to WHO Moment 3 (after body fluid exposure risk). This method comprises the same HH moments as the WHO standard, is more adapted to the nursing home setting, is easier to remember (one slogan), and is easier to visualize.

After explanation of the HH moments and reiteration that the participants are now expected to follow the rules for HH, the participants had time to ask questions. The next step was to ask the participants to pick a HH goal that would receive extra attention. This group goal was a moment that they thought was attainable and immediately implementable. The main reasons for creating a goal were to reflect upon what was just learned, create a sense of ownership, and create team commitment. All goals mentioned during the day’s session were printed on a small poster and hung in the nurses’ office to act as a reminder.

A larger, colorful poster was presented. Participants were told that different posters would come every month and asked where they would like the posters to hang so that they felt ownership of the project.

To encourage e-Learning participation, participants received a nurse’s watch (which you can pin on your clothing) after completion of the e-Learning. They also left the meeting with an immediate reward, since they left with a small bottle of hand sanitizer to be used during the e-Learning. This was done to create a positive association with HH. After Lesson 1, the management-level contact(s) were informed in person and by mail of any pertinent staff comments so that they could consider making system or facility changes.

Between Lesson 1 and Lesson 2, a senior nursing home manager presented the newest rules for personal hygiene to the nurses and nurses’ aides. Materials were made available to assist the manager with the presentation, including a picture of an agar with bacterial growth caused by a ring and a poster displaying personal hygiene rules. Nurses and nurses’ aides were informed of their organization’s disciplinary consequences if they did not adhere to the personal hygiene rules.

Lesson 2 lasted 30 minutes and was usually combined with Lesson 3 to create one lesson of 50 minutes. The main goal of the second lesson was to remove the barriers that nurses experience when trying to perform HH. Each participant was given a sheet with 28 stickers representing 13 different barriers. There were 2 blank stickers, allowing participants to write down any additional barriers. The stickers represented 4 themes, namely facilities, forgetting, choosing not to do HH, and the telephone. The barriers were identified through literature, interviews, and observations.

Sheets of paper were hung on the walls, one sheet for each of the 4 elements of the slogan (Room In, Room Out, Before Clean, After Dirty). Participants were asked to place one sticker on each piece of paper representing the main reason that he or she did not perform HH at that moment. This system facilitates an organizational diagnosis of HH impediments. Once the stickers were placed, the most prevalent barriers were discussed. Group discussions resolved barriers by designing new coping strategies, cues to action, and environmental changes. The barrier analysis with solutions was in turn discussed with the nursing home manager so that any necessary system or facility changes could take place.

During Lesson 3, participants learned the correct execution of HH through active participation. Using gloves and paint, participants saw which parts of their hands they missed when washing them incorrectly and that fluids, bacteria, and viruses can get on hands during glove removal. They also learned the correct HH procedure. Although the WHO promotes a 6-step method [25], wrist rubbing was added since this area can easily be contaminated when removing gloves. After the third lesson, management was informed of any participant feedback that could influence HHC.

#### E-Learning

The e-Learning served two purposes: It allowed nurses and nurses’ aides who were unable to attend the live lessons to gain HH knowledge, and it provided reinforcement of these lessons. The e-Learning consisted of an introduction and 7 lessons. The themes of the lessons were the resident’s perspective, how to wash and disinfect your hands, when to execute HH, HH in combination with sterile activities, time-saving work habits, glove use, and social aspects of HH. Videos modelled knowledge, guided practice, and promoted active learning by encouraging participants to scrutinize videos.

After viewing the introduction, the participant was invited every other week to complete the next lesson. This method provided participants with regular reminders to perform HH. Each lesson lasted 5-10 minutes and ended with a quiz to reinforce the message. After completing the entire e-Learning, the participant received a certificate and a nurse’s watch.

#### Posters, Photo Competition, and an Arts and Crafts Project

To reinforce the message, 3 supplementary components were developed, namely posters, a photo competition, and an arts and crafts project. The posters acted as reminders and included large pictures of hands and the text: “Did you remember to wash your hands?” (*Vergeet je niet je handen te wassen?*). The posters were designed to be visually appealing with a cheery image so that they could be placed in living areas. New posters were distributed monthly over a 10-month period so that the message would repeatedly capture attention. Of these posters, 5 came from the photo competition and the arts and crafts project.

Participants were invited to submit a photo for the photo competition. The idea behind this activity was to get nurses to think about HH in diverse situations, including outside the workplace. The photo submission needed to contain pictures of hands. The winners of the 3 best photos received a gift certificate. Their photos were used for 3 of the monthly posters.

Additionally, nursing homes received a package of information containing instructions on implementing a hand-related arts and crafts project with the residents. This activity had 3 goals: to create a training moment for the residents to learn when to perform HH, to inform residents that the staff is paying more attention to HH, and to again remind staff to perform HH. The 2 most appealing pieces of art were turned into 2 of the monthly posters.

### Strategies to Improve and Monitor Adherence to Protocols

While the researcher used persuasive communication to convince nursing home management to allow the entire nursing staff to participate in all 3 lessons, we assumed that not everyone would attend. Intervention adherence was documented. Attendance at the HH lessons and e-Learning lessons was recorded. Additionally, attendees were asked in the process evaluation if they received information about personal hygiene policy and if they saw HH posters hanging in the nursing home.

### Outcomes

#### Primary Outcome Measure

HHC is the primary outcome measure. HHC is defined as the number of times that HH is performed at an HH opportunity (according to the WHO’s 5 moments of HH), divided by the total number of times that it should be performed, expressed as a percentage. We only documented HH as compliant if hand sanitizer or soap, water, and a paper towel were used. Compliance was measured through live observations, still considered the gold standard, even though there is a risk of observer bias and the Hawthorne effect [26,27]. There were 4 registered timepoints (Table 2).

**Table 2:** Timeline of the study.

		Study period									
		Recruitment	Baseline	Randomization	Post-allocation						Close-out
Timepoint		Mar-Sep 2016	Oct 2016	Nov 2016	Dec 2016	Jan 2017	Feb 2017	Mar-Apr 2017	May 2017	Oct 2017	Nov-Dec 2017
**Recruitment**											
	Eligibility screening	X	—^a^	—	—	—	—	—	—	—	—
	Signed commitment	X	—	—	—	—	—	—	—	—	—
	Randomization	—	—	X	—	—	—	—	—	—	—
**Intervention (fixed intervention arm)^b^**											
	Meeting with management	—	—	—	X	—	—	—	—	—	—
	Lesson 1	—	—	—	—	X	—	—	—	—	—
	Lessons 2 & 3	—	—	—	—	—	—	X	—	—	—
	E-Learning^c^	—	—	—	—	X	X	X	X	X	—
	Posters^c^	—	—	—	—	X	X	X	X	X	—
**Assessments**											
	Structured interview	X	—	—	—	—	—	—	—	—	—
	Compliance observations	—	X	—	—	—	X	—	X	X	—
	Illness registry^c^	—	X	X	X	X	X	X	X	X	—
	Microbiology samples	—	X	—	—	—	X	—	X	—	—
	Process evaluation	—	—	—	—	—	—	—	—	—	X
	Close-out questionnaire	—	—	—	—	—	—	—	—	—	X

^a^Not applicable.

^b^For the conditional intervention arm, the intervention timeline was dependent upon the month an outbreak occurred.

^c^Continuous intervention exposure or measurement.

#### Secondary Outcome Measure

The incidence of gastroenteritis, influenza, assumed pneumonia, MRSA, and urinary tract infections in the nursing home residents is the secondary outcome measure. Nursing home staff recorded these infectious diseases on a weekly basis, along with infectious disease outbreaks. The McGeer criteria were used to define the infectious diseases [28].

#### Additional Outcome Measures

Additional outcome measures included the presence of norovirus, rhinovirus, and *Escherichia coli* on 3 types of surfaces in the nursing home. Norovirus is a common viral gastrointestinal pathogen, rhinovirus is a common respiratory pathogen, and *E. coli* is a common bacterial indicator of fecal contamination of the physical environment. To measure the presence of these pathogens, microbiology samples were taken with wipes and sent to the laboratory for analysis. Samples were taken during the first 3 timepoints for the primary outcome.

Hand-related personal hygiene compliance was also documented as an additional outcome measure. This was measured according to Dutch guidelines [24]. A nurse was considered compliant if he or she did not have long nails, acrylic nails, or polished nails and did not wear a ring, bracelet, wristwatch, brace, or long sleeves. Personal hygiene was noted for every nurse who was observed for HHC. Compliance is defined by the percentage of personal hygiene–compliant nurses, divided by the total number of nurses observed. Hand-related personal hygiene compliance was documented at the same timepoints as the primary outcome measure.

### Timeline

The recruitment period lasted from March through September 2016 (Table 2). The trial began with baseline measurements of HH, personal hygiene, and environmental sampling in October 2016 (baseline). At this point, nursing homes began submitting a weekly disease incidence report of the illnesses mentioned earlier. After the baseline measurements, nursing homes were allocated to 1 of the 3 study arms. For the fixed intervention nursing homes, this was followed by a meeting with management, the first lesson, presentation of the e-Learning, start of monthly posters, and announcement of the photo competition. After the first lesson, the first follow-up observations occurred at the fixed intervention and control nursing homes 3 months after baseline. This was followed by the second and third HH lessons and the dissemination of information about the arts and crafts project at the fixed intervention nursing homes. 6 months after baseline, both the fixed intervention and control nursing homes were observed again. The last observation occurred at the fixed intervention and control nursing homes 12 months after baseline.

After randomization, individual conditional intervention nursing homes followed the same schedule as the fixed intervention nursing homes, but only after an outbreak occurred. Preliminary analyses of the outcome measures were performed after every round of observations. All data were collected by December 2017. This study will be completed in 2020.

### Sample Size Calculation

The HH intervention was expected to increase HH compliance from 35% pre-intervention to 50% post-intervention. The sample size was calculated based on 80% power with a two-sided α of .05, taking into account the clustering of observations within nursing homes and assuming a heterogeneity between nursing homes of 0.4. It was determined that a sample size of 45 nursing homes would be sufficient, with 15 nursing homes participating in each arm. Since we could not assume that all nursing homes in the conditional intervention arm would have an outbreak during the study period, the goal was to have a minimum of 25 nursing homes in this arm.

We aimed to evaluate 2 units at each nursing home and to observe 3 nurses in each unit for a maximum of 2 hours each. This equates to 12 hours of observation per nursing home per observation round, in which we expected to observe 75 HH opportunities, equally divided over the 5 moments of the WHO. We therefore expected approximately 1125 opportunities per arm per observation round.

### Blinding

Blinding the researcher to the intervention arm was not possible in this trial because the researcher also taught the lessons. The nurses were blinded by giving distinct names to the lessons (The New Way of Working) and the observations (HANDSOME), so that they appeared to be different projects. Furthermore, nurses were told that the observers were registering the frequency of health care activities. HH observers were not informed which nursing homes were receiving the intervention, although they may have noticed HH posters from the intervention while observing.

### Data Collection Instruments

Before the first observation, nursing home unit managers were interviewed in person or over the telephone. A baseline questionnaire was used to gain more insight into the background characteristics of each individual unit, such as the number of employees, brand of HH products, and type of care provided by the unit.

We designed a tablet-based observation app to measure HH and hand-related personal hygiene. The registration events were based on the 5 moments of HH, as determined by the WHO and Dutch guidelines for personal hygiene [16,24]. Hand-related personal hygiene was recorded once for every observed nurse per observation day.

When documenting HH, a distinction was made between the use of hand sanitizer or combination of water, soap, and paper towel. If neither method was used at an opportunity or if the water-soap-paper towel combination was missing one element, then the HH opportunity was considered “missed.” To be considered compliant, HH needed to happen in the same room in which the action occurred. The only exceptions to this rule were if a nurse brought a resident to another room, a nurse carried something soiled, or no door needed to be opened before leaving the room. In these cases, HH should have taken place at the end of the action.

Compliance to the 5 moments of the WHO was broken down into submoments, giving more insight into the frequency of and compliance at submoments (Table 3). Three additional activities that potentially facilitate pathogen transmission were registered separately, namely the preparation and serving of food and medication, taking gloves to use for non-resident related activities, and social contact. HHC related to food and medication activities was documented since this could be considered a clean procedure (Moment 2). HH before taking gloves for non-resident related activities was noted because taking gloves without first performing HH may contaminate other gloves from the same box [29]. According to the WHO guideline for long-term health care, HH is not required during social contact, even though it does involve hand contact and thus potentially facilitates pathogen transmission [16]. We therefore recorded the number of times that this occurred. We defined social contact as patting the shoulder/knee, shaking hands, patting a hand, and hugging.

**Table 3:** Moments and submoments for hand hygiene compliance documentation.

Moment		Submoment
Moment 1 (before touching a patient)		Washing or providing perineal care in own room, providing perineal care at the toilet, other care, and after the use of a mobile phone, tablet, or computer during resident contact (during Moment 1 activities)
Moment 2 (before clean/aseptic procedure)		Catheter care, wound care, injection, feeding tube care, colostomy care, pain pump care, eye drops, tracheostomy tube care, mucous suction, other invasive care, and after the use of a mobile phone, tablet, or computer during resident contact (during Moment 2 activities)
Moment 3 (after body fluid exposure risk)		Invasive care, removing bedding, washing/cleaning the resident in own room, helping resident at the toilet, other (body fluid of a resident), own body fluid, helping animals, and before the use of a mobile phone, tablet, or computer during resident contact (during Moment 3 activities)
Moment 4 (after touching a patient)		Resident care and before the use of a mobile phone, tablet, or computer during resident contact (during Moment 4 activities)
Moment 5 (after touching a patient’s surroundings)		No submoments
**Additional potential moments for pathogen transmission**		
	Before using gloves (not patient-related)	No submoments
	Before food and pills	Preparing or administering medicine, preparing food, serving food, helping with eating, and washing the resident’s hands before eating
	Social contact	Pat on the shoulder, shaking hands, touching a hand, and hugging

Once the observations were finished with one nurse, the observer reset the app for the observations with the next nurse. Personal hygiene compliance was only registered one time per nurse.

The residents’ infectious disease occurrence was recorded by staff. Each unit received a notebook in which a designated person (nurse, team leader, or geriatrician) recorded the weekly incidence of gastroenteritis, influenza, assumed pneumonia, MRSA, urinary tract infections, and an outbreak. The nursing home was free to decide who was responsible for the reporting. We only collected anonymized patient data. Definitions of the illnesses were given in the notebook to promote homogeneity in reporting. Weekly reports were sent to the researcher via email or WhatsApp.

Microbiology samples were collected at baseline, 3 months after baseline, and 6 months after baseline (Table 2). Samples were taken from a communal table, a communal toilet, and the computer mouse and keyboard. The qualitative molecular detection technique quantitative polymerase chain reaction was used to detect viral indicator organisms and *E. coli*. The wipes used in this process do not supply quantitative results, but they make it possible to cover a larger surface area than with swabs, enhancing the sensitivity.

A process evaluation occurred after the intervention. Every nurse who attended at least one live lesson or started the e-Learning received an email with a link to a process evaluation questionnaire. They were asked questions to measure fidelity at the unit and their opinion about different aspects of the intervention.

After the intervention was completed, a senior nursing home manager participated in a close-out questionnaire to assess system changes or infection prevention programs that may have affected HHC during the study period.

### Measuring Compliance: Training and Planning

Independent observers were trained to observe HHC using an adapted training method from an HHC study in Dutch hospitals [30]. Observers were primarily nurses and doctors in training. These observers were trained over a period of 2-3 days using videos, case studies, and live observations at 2 nursing homes. The training ended with an examination using videos from Hand Hygiene Australia [31]. The observers also received training in collecting microbiological samples.

Observers documented nurses’ HHC at the nursing home from 8:00 am to 2:00 pm. The objective was to observe a minimum of 3 nurses, each for a maximum of 2 hours.

### Promoting Participant Retention

If a nursing home considered stopping the intervention, it was encouraged to continue the program through persuasive communication. If the nursing home refused to follow the protocol, the researcher had the option to withdraw the participant from the program. If the nursing home dropped out of the intervention, management was still asked to answer questions in the close-out questionnaire.

### Data Management and Dissemination

Data were collected in different ways. Background information about the nursing homes and information from the close-out questionnaire were collected during interviews and from forms sent from the nursing homes. This information was entered in an Excel (Microsoft Corp, Redmond, WA) document. Weekly infectious disease incidence reports were similarly entered in an Excel document by a dedicated staff member. All compliance data were entered in an app and downloaded into Excel documents. Compliance data will be cleaned in SPSS version 25 (IBM Corp, Armonk, NY). The results of the microbiology samples were entered in an Excel document. Information from the process evaluation was gathered with an online survey and downloaded into SPSS. HHC and protocol adherence results were disseminated to participating nursing homes in personalized reports. The results of the study will be made available to the wider community in scientific publications. Data will be managed and archived according to the Quality Manual of the Department of Public Health, Erasmus MC, University Medical Center Rotterdam. Researchers may request access to the data from the chair of the Department of Public Health, Erasmus MC, University Medical Center Rotterdam.

### Statistical Methods

The various outcomes of the trial (primary, secondary, and additional) will be analyzed separately according to the specific research hypotheses (Table 4).

**Table 4:** Statistical methods

Outcome	Hypothesis	Outcome measure	Methods of analysis
Primary: hand hygiene	Improvement is higher in the intervention arms than the control arm.	Hand hygiene compliance (binary)	Multilevel logistic regression
Secondary: infectious disease incidence	There will be a lower disease incidence in the intervention arms than in the control arm.	Infectious disease incidence (binary)	Multilevel logistic regression
Additional: presence of norovirus, rhinovirus, and *Escherichia coli*	There will be a lower detection rate of microorganisms on surfaces in the intervention arms than in the control arm.	Proportion of samples positive for norovirus (genogroups I and II), rhinovirus (continuous), and *Escherichia coli*	Multilevel loglinear regression
Additional: personal hygiene	Improvement is higher in the intervention arms than in the control arm.	Personal hygiene compliance (binary)	Multilevel logistic regression

### Ethics Approval and Consent to Participate

Ethical approval for the study was waived by the Medical Ethics Review Committee of the Erasmus MC (no.58158). Any significant changes to the study protocol were communicated to the Medical Ethics Review Committee. All changes were communicated to the participants, steering committee, and study sponsor. Consent to participate is not relevant in this study, since we did not collect any patient information. No identifying information about the nurses was collected. All collected data will be anonymized before publication to protect the privacy of the nursing home and nursing home staff. Data sets will be anonymized according to our quality manual and data management plan.

## Results

The study was funded in September 2015. Medical ethical approval was waived in August 2016. Data collection started in October 2016 and was completed in October 2017. In total, 124 nursing home units were recruited in 62 nursing homes. Of these, 116 units were allocated: 36 to the fixed intervention arm, 50 to the conditional intervention arm, and 30 to the control arm. Data analysis is ongoing, and the first results are expected to be published in 2020.

## Discussion

The HANDSOME study was created to increase HHC in nursing homes. We took this opportunity to not only look at HHC but also to investigate a secondary outcome of the incidence of gastroenteritis, influenza, assumed pneumonia, MRSA, and urinary tract infections in the nursing home residents. The presence of norovirus, rhinovirus, or *E. coli* on nursing home surfaces was also documented, creating the opportunity to triangulate with HHC and infectious disease incidence. We also documented hand-related personal hygiene compliance.

The HANDSOME intervention was developed specifically for the nursing home setting. It used a blended learning model to reach as many nurses as possible. HANDSOME reframes the WHO’s HH moments so that they are understandable and easily recalled in a nursing home setting. We created the slogan “Room In, Room Out, Before Clean, After Dirty,” which incorporates the WHO framework for HH. It specifically takes into account that most health care actions occur in the residents’ bedrooms, social contact is excluded from the HH rules in nursing homes, and it is only feasible to consider the resident’s room (or that portion of the room that belongs to him or her) as the resident’s surroundings.

“Room In, Room Out” is a concept that has been used before in HH policies, mostly with the terms “Wash In, Wash Out” [32,33]. The “Wash In, Wash Out” method is problematic for various reasons. It inherently neglects HH before an aseptic procedure and after contact with bodily fluids. Additionally, as demonstrated by Sunkesula et al [34], the health care worker would often be expected to do unnecessary HH when using the “Wash In, Wash Out” method since health care workers often do not touch patients in the patient’s room. Furthermore, “Wash In, Wash Out” inherently emphasizes hand washing and ignores the benefits of using hand sanitizer. We address these problems by using the terms “Room In, Room Out, Before Clean, After Dirty” and teaching participants in the lessons and e-Learning that they do not need to perform HH in a resident’s room if they do not touch the resident or the resident’s surroundings and they can omit “Room In” if they only touch the resident’s surroundings without touching the resident.

Our observational method should also give more insight into HHC moments. Our study is one of the few that looks specifically at the separate moments and submoments of the 5 WHO moments. This way, we can gain better insight into which health care actions occur most frequently in nursing homes and which moments need the most attention to attain a higher HHC and less illness. We also expect to gain more insight into barriers for each HH moment. During the second lesson, participants were asked to specify barriers experienced during the different HH moments.

This study should add to the body of evidence that HHC is suboptimal in nursing homes and can be significantly improved through an intervention. We also expect to gain insight in personal hygiene compliance in nursing homes. Another strength of this study is that it created an aggregate register of residents’ infections. Although there are some data about HAIs in nursing homes, most nursing homes only register illness in individual dossiers [1]. This study collected data about infection incidence using the same definitions as the National Institute for Public Health and the Environment in the Netherlands so that the data can be compared [35]. This could add more insight and help form the agenda to avoid unnecessary illness. We believe that this is also one of the first studies to systematically sample nursing home surfaces for various viruses and bacteria in order to study the potential added value as an alternative method to monitor HHC.

Another novel aspect of our intervention is that we may discover if an intervention is more successful at a random point in time or after an infectious disease outbreak. We should create more insight into when HH interventions should be implemented.

This study also has limitations. Since we used the gold standard of measuring HHC, observers directly observed nurses giving care. This may have caused Hawthorne or observer bias. A second limitation is that nursing homes were not required to send every nurse to the lessons, conceivably causing a significant variation in compliance to the protocol. Another limitation could be that observers were able to guess which nursing homes received the intervention, since these nursing homes had HH posters from the intervention hanging on the walls, which may unconsciously have influenced their observations. Last, we only observed HH at organizations with at least 3 nursing homes. This study therefore does not necessarily reflect HHC at smaller organizations.

Considering that there are few studies that have rigorously investigated the WHO’s recommendations for HH, HANDSOME will provide needed insight into HH in nursing homes. The results from this study could help in creating more refined and successful HH interventions in the future. Future interventions can focus on the moments that are more often missed.

## Abbreviations

HAI: health care–associated infection.
HH: hand hygiene.
HHC: hand hygiene compliance.
WHO: World Health Organization.
